# Weight Bias Internalization Among Adolescents Seeking Weight Loss: Implications for Eating Behaviors and Parental Communication

**DOI:** 10.3389/fpsyg.2018.02271

**Published:** 2018-11-21

**Authors:** Rebecca M. Puhl, Mary S. Himmelstein

**Affiliations:** ^1^Department of Human Development and Family Studies, Rudd Center for Food Policy and Obesity, Hartford, CT, United States; ^2^Mary Himmelstein, Rudd Center for Food Policy and Obesity, Hartford, CT, United States

**Keywords:** internalization, weight, youth, eating behavior, parent-child relations

## Abstract

**Background:** Emerging evidence has demonstrated a high prevalence of weight bias internalization (WBI) among adults, as well as consistent links between internalization and adverse psychological and physical health. However, research examining WBI in youth and its impact on their health is scarce, especially among youth seeking weight loss treatment who may be particularly vulnerable to weight stigma from peers and parents. To address this research gap, the present study assessed WBI in a weight loss treatment-seeking sample of adolescents, examining associations between internalization and adolescents’ eating behaviors and parental weight-related communication.

**Methods:** Adolescents (*N* = 148, *M*_age_ = 15.97 years), completed online self-report measures to assess WBI (using the modified version of the WBI Scale), body weight, binge eating, eating as a coping strategy, and weight teasing from peers and family members. Adolescents also reported on the frequency of parental comments about body weight, parental dieting, and parental encouragement of adolescent dieting.

**Results:** Adolescents expressed a high mean level of internalized weight bias (*M* = 5.45, *SD* = 0.88). Higher levels of internalization were observed across increasing body weight categories; no differences were observed for gender or history of weight teasing. WBI was significantly higher among adolescents who reported binge eating and eating to cope with distress. Regression analyses showed that weight-related comments from mothers (but not fathers) significantly predicted adolescents’ WBI (including frequency of mothers’ comments about adolescents’ body weight, comments about their own body weight, and encouragement of their adolescent to diet), as did increased dieting frequency among mothers.

**Conclusion:** The present study provides novel insights to the scant literature on WBI in youth. Findings indicate that WBI is high in both girls and boys engaged in weight loss, and is associated with maladaptive eating behaviors, higher frequency of maternal dieting, and mothers’ comments about body weight. These findings have important clinical implications for youth and families engaged in weight loss treatment, and underscore the need for research to clarify adverse effects of internalization on weight-related health in youth and to better understand the role that parental weight communication may have on adolescents’ internalization.

## Introduction

Children and adults with higher body weight are vulnerable to societal bias and stigma because of their weight (Puhl and Heuer, 2009). Individuals who experience weight bias face societal devaluation, negative stereotypes, prejudicial attitudes, and unfair treatment from others because of their weight. For youth and adolescents, these experiences most often occur in the form of weight-based teasing, bullying, and victimization from peers and family members. Recent studies of adolescents, parents, and educators consistently show that weight-based bullying is one of the most prevalent forms of youth bullying in our society (Puhl et al., 2011, 2013a, 2016 ; Bradshaw et al., 2013). Youth with obesity appear to be particularly vulnerable to these experiences, with some research indicating that among adolescents seeking treatment for obesity, as many as 90% report weight-based victimization from peers, and 60% from family members (Puhl et al., 2013b; Puhl and Himmelstein, 2018). In addition, over a third of these youth report that weight-based victimization has persisted for at least 5 years (Puhl et al., 2013b). With increasing research attention to the commonality of weight-based victimization among diverse groups of youth (Bucchianeri et al., 2013), studies have also examined the impact of these experiences on quality of life for children and adolescents; over a decade of evidence has demonstrated links between weight bias and negative health consequences for youth (Goldfield et al., 2010; Puhl and Luedicke, 2012; Bucchianeri et al., 2014; Warkentin et al., 2017), including multiple indices of psychological distress and poor physical health, and longitudinal associations between weight-based teasing from peers and family members and adverse weight-related health in late adolescence and adulthood (Eisenberg et al., 2006; Haines et al., 2006; Quick et al., 2013; Hunger and Tomiyama, 2014; Puhl et al., 2017b).

Adding complexity to this problem is that individuals who are vulnerable to experiences of weight bias may also internalize bias and direct it toward themselves. Known as *weight bias internalization* (WBI) (Durso and Latner, 2008; Pearl and Puhl, 2018), this process of self-stigma involves becoming aware of negative stereotypes about one’s stigmatized identity, agreeing with these stereotypes, applying stereotypes to oneself, and engaging in self-devaluation because of one’s stigmatized identity (Corrigan et al., 2006). As a result, this process leads people to engage in self-disparagement and self-blame in response to weight bias expressed and enacted by others.

Internalized weight bias has received increasing research attention in recent years, especially in adults. Recent evidence with national samples of Americans suggest that as many as 40% of adults with overweight and obesity have internalized weight bias, and 20% express high levels of internalization, with more women than men reporting high levels (Puhl et al., 2017a). In addition, an amassing literature has demonstrated consistent links between internalization and adverse psychological and physical health. The first systematic review of this literature published in 2018 examined 74 studies assessing the relationship between WBI and health in both community and treatment-seeking samples of adults (Pearl and Puhl, 2018); results showed that WBI was consistently related to adverse mental health indices including depression, anxiety, poor body image, health related quality of life, and disordered eating behaviors. While fewer studies have examined links between WBI and physical health, clear associations were observed between WBI, increased severity of obesity, lower self-efficacy for engaging in healthy behaviors, and worse dietary adherence (Pearl and Puhl, 2018). Furthermore, mediational analyses indicate that WBI explains the relationship between experiences of weight bias and adverse health indices (e.g., eating pathology) (Durso et al., 2012a; O’Brien et al., 2016). Collectively, this evidence highlights that internalized weight bias is both common and potentially damaging for health, independent of body weight and experiences of stigma.

In contrast to the rapidly emerging literature on WBI in adults, very little is known about WBI in youth. Of the few studies in this area, two cross-sectional studies have focused on validating an existing measure of WBI frequently used in adult populations (the Weight Bias Internalization Scale; WBIS) in samples of adolescents, including a U.S. sample of 57 adolescents (80% girls) seeking bariatric surgery (Roberto et al., 2012), and testing a modified version of the WBIS in a German sample of 191 adolescents (51% girls) seeking weight loss treatment (Ciupitu-Plath et al., 2017). These studies showed that the WBIS is an appropriate and suitable measure for assessing WBI in adolescents seeking treatment for obesity, and that internalized weight bias was related to poorer psychological functioning, such as depression, anxiety, psychiatric problems, disordered eating, binge eating, poor body image and lower self-esteem. Of note, the U.S. study found no gender differences in levels of WBI, while the German study observed higher WBI in girls compared to boys. A third validation study focused on a different measure of WBI (the Weight Self-Stigma Questionnaire; WSSQ) in a sample of 156 Canadian (French-speaking) adolescents, also demonstrating suitability of this measure and significant correlations with disordered eating, depression, anxiety, and poor self-esteem, but not BMI (Maïano et al., 2017).

The only prospective research examining WBI in youth is a German study that followed a community-based sample of 1,047 children (ages 7–11 years) for 2 years (Zuba and Warschburger, 2017). Findings showed that WBI mediated the relationship between BMI and psychosocial problems (such as restrained eating, emotional and conduct problems), and that this pattern of findings held regardless of children’s gender or weight status. While baseline levels of WBI were higher in children with overweight and obesity compared to children with lower body weight, no gender differences were found for associations between WBI and BMI or other psychological health indices.

While these several studies provide initial evidence that WBI may begin in youth and potentially contribute to similar adverse health consequences that have been documented in adults, there is a clear need for more research in this area and key questions remain. First, given high rates of weight-based victimization reported by adolescents with obesity, it seems especially warranted to study WBI and its associations with health behaviors in this vulnerable population. Second, no research to date has examined whether different sources of weight-based victimization (e.g., teasing from peers versus family members) have differential associations with internalized weight bias in youth. Third, although previous research indicates that parental comments and/or teasing about weight can contribute to emotional distress and adverse eating behaviors in adolescents (Kluck, 2010; Neumark-sztainer et al., 2010; Bauer et al., 2013; Mustillo et al., 2013), no research has examined these types of parental factors in the context of WBI in youth. Many parents report engaging in weight-focused communication with their adolescents (Berge et al., 2015; Winkler et al., 2018), and many adolescents report that their parents make critical or judgmental comments about their weight (Keery et al., 2005; Fulkerson et al., 2007; Lo et al., 2009; Neumark-sztainer et al., 2010; Berge et al., 2016). “Thus, as part of research efforts to better understand the nature and extent of WBI in youth, it is important to determine whether relevant parental factors contribute to this issue, such as whether youth are more likely to internalize weight bias if they are exposed to parental weight comments. To begin to address these understudied issues, the present study assessed WBI in a weight loss treatment-seeking sample of adolescents. Specifically, this study explored the following research questions: (1) is WBI positively related to binge eating and eating to cope with stress, and (2) is WBI positively predicted by sources of weight-based victimization (peers and family members), and adolescent reports of parental weight-related comments (parental comments about weight and dieting).

## Materials and Methods

### Study Participants

Participants in the present study were comprized of adolescents (*N* = 148) who were enrolled in a national commercial weight loss camp (Camp Shane) in 2017. Camp Shane focuses on behavioral weight loss and weight loss maintenance for youth and young adults, and requires that all campers have a documented medical history and physician’s appointment in order to attend camp. The camp has six locations in the U.S. (Arizona, California, Georgia, New York, Texas, and Wisconsin), and data collection occurred between April and July of 2017. Upon camp registration, parents of adolescents between the ages of 13–18 years at each camp location were provided with a two-page permission form describing the study, inviting their son or daughter to participate in the present study. Parents indicated their permission on this form if they consented to allow their adolescent to participate, and this form was submitted online by parents as part of all other required camp registration forms. Parents who indicated consent were then emailed a weblink to an online survey (hosted by Qualtrics.com) to share with their adolescent so that he/she could complete the survey. When adolescents clicked on the weblink, they were presented with an introductory webpage providing information about the study’s purpose and procedures; it was made clear on this page that the survey aimed to understand adolescents’ experiences and perspectives about weight-based teasing and bullying. Only after reading this page, and providing assent to participate (by clicking an icon indicating their agreement that they had read this information and accepted the conditions of the study) could adolescents access the survey. Participation was voluntary, and it was made clear to participants that they could stop the survey at any time without any consequences. Adolescents were asked to complete the survey online in a private setting (e.g., at home). Following survey completion adolescents received a gift card to a national online retailer.

In total, there were 459 campers registered across all camp locations, of which 309 adolescents were in the eligible age range to participate in the study; the response rate was 48% (*N* = 148). Compared to the total number of campers (*M*_age_ of 15.26 years, *SD* = 1.63), adolescents who chose to participate in the present study were slightly older (*M*_age_ of 15.87, SD 1.25). In addition, study participants were primarily from the New York (43.9%) and California (31.8%) camp locations, followed by Georgia (12.2%), Texas (6.1%), Arizona (4.7%), and Wisconsin (1.4%). The study protocol was approved by the University of Connecticut’s Institutional Review Board and parents of all adolescents provided written informed consent in accordance with the Declaration of Helsinki.

### Survey Measures

#### Participant Demographic Characteristics and Body Weight

Adolescents reported their age, gender, race/ethnicity, and current height and weight. BMI percentiles for age and sex were calculated and categorized using tools provided by the Centers, 2012. Weight categories corresponding to BMI percentiles include healthy weight (≥5th to <85th percentile), overweight (85th to <95th percentile), and obesity (≥95th percentile).

#### Weight Bias Internalization

Adolescents completed the modified, validated version of the Weight Bias Internalization Scale (WBIS-M), a widely used measure to assess WBI (Pearl and Puhl, 2014; Durso et al., 2016). The WBIS has been used previously in adolescents with obesity seeking weight loss (Roberto et al., 2012), and assess the degree to which people apply negative weight-based stereotypes to themselves and judge themselves negatively due to their weight. The original WBIS-M consists of 11 items; in accordance with recent research on the psychometrics of this measure (Durso et al., 2016; Lee and Dedrick, 2016), the first item was dropped, resulting in a 10 items-scale in which adolescents rated their extent of agreement with statements such as “I don’t feel that I deserve to have a really fulfilling social life, because of my weight.” Items were rated on a Likert scale from 1 (strongly disagree) to 7 (strongly agree), with higher scores indicative of higher levels of internalized weight bias. Cronbach’s alpha in the present sample was α = 0.86.

#### Weight-Based Victimization

Perceived weight-based teasing and bullying was assessed with two previously established yes/no questions tested in samples of adolescents: “Have you ever been teased or bullied because of your weight by your peers or other students?” and “Have you been teased or treated unkindly by family members because of your weight? (Neumark-Sztainer et al., 2006, 2012; Puhl et al., 2013b)” These questions were slightly adapted from two questions previously used and widely published from Project EAT, a longitudinal cohort study examining weight-related experiences of adolescents. If participants indicated “yes” to either or both of these questions, they were prompted with additional questions about the frequency of these experiences happening in the past year (5-point scale ranging from *never* to *very often*), with higher scores indicating a greater frequency.

#### Eating as a Coping Strategy

Eating to cope with stress was measured using the validated 5-item Coping Subscale from the Motivations to Eat Measure (Jackson et al., 2003), which has been used in adolescent populations (Thogersen-Ntoumani et al., 2009). Participants were asked how often they eat because they are depressed or sad, feel worthless or inadequate, as a way to help them cope, as a way to comfort themselves, or as a way to avoid thinking about something unpleasant or to distract themselves. Items were rated using a 5-point scale of 0 (*almost never*) to 4 (*almost always or always*), with higher scores indicating a higher frequency of using food to cope with stress. Cronbach’s alpha in the present sample was α = 0.61.

#### Binge Eating

Binge eating was measured using four previously established and validated questions (Yanovski, 1993) used in large samples of adolescents (Neumark-Sztainer et al., 2004, 2006), assessing the presence of binge eating in the past year (yes/no), with or without loss of control (yes/no), frequency of binge eating with loss of control (4-point scale from *every day* to *less than once per month*), and distress over binge eating (4-point scale from *not at all* to *a lot*). Following previous research (Neumark-Sztainer et al., 2006), these items were combined to determine a severity score, with lower scores indicating more binge eating severity. Specifically a score of 1 represented binge eating episodes occurring at least once per week with loss of control and emotional distress in response to overeating; a score of 2 reflected binge eating with loss of control and at least some distress over binge eating; a score of 3 equated to the presence of binge eating but no loss of control and no distress over binge eating; and a score of 4 equated to no binge eating.

#### Parental Comments About Weight and Dieting

Questions assessing adolescents’ perspectives of parental comments about weight and dieting were modified from previously used survey measures used with adolescents in Project EAT (Neumark-sztainer et al., 2010; Bauer et al., 2013). Adolescents were asked “how often does your mother make comments to you about you weight” and “how often does your father make comments to you about your weight?” Responses to these questions were rated on a 5-point scale from *never* to *very often*. Participants were then asked to indicate how much their mother and father each talk about his or her own weight, engage in dieting (defined as “diets to lose weight or keep from gaining weight”), and encourages the adolescent to diet. These questions were rated on a 4-point scale from *not at all* to *very much*.

### Analytic Strategy

Independent samples *t*-tests assessed differences in WBI as a function of sex, and a one-way ANOVA assessed differences in WBI as a function of BMI categories based on BMI percentiles for age and sex. We tested for sex × BMI category interactions on WBI using 2 × 2 ANOVA, but none emerged. For parsimony we only report the independent *t*-test and one-way ANOVA below. Linear regressions assessed the relationship between WBI and binge eating severity as well as WBI and eating as a coping strategy for dealing with stress. A linear regression examined relationships among the presence of WBV (family, peers), frequency of weight-teasing (family, peers), frequency of parental comments about weight, frequency of parental encouragement to diet, frequency of parental comments about their own weight, and frequency of parental dieting. All regression models controlled for participant sex (male as reference group), BMI percentile for age and sex, age, and race (white as the reference group).

## Results

### Sample Characteristics

Table 1 displays demographic and weight-related characteristics of participants. The total sample included 50% boys and 50% girls, and 90% were White. The mean BMI percentile of adolescents was 27.06 (*SD* = 4.39); the distribution of weight categories according to BMI classifications included 34.5% in the obese range, 37.2% in the overweight range, and 28.4% in the healthy weight range (reflecting adolescents who had achieved significant weight loss and were returning to camp for weight loss maintenance). Almost all participants (93.9%) reported experiencing weight-based bullying from peers, and 60.1% reported being teased about their weight from family members.

**Table 1:** Sample characteristics (*N* = 148).

Variable	Range		*M*	*SD*	α
Age	13	18	15.97	1.25	
BMI	18	40	27.06	4.39	
BMI percentile	14	99	85.92	16.69	
Weight bias internalization mean	2	7	5.45	0.88	0.86

	***N***	%			

Race/ethnicity
White	134	90.5			
Black	3	2.0			
Asian	4	2.7			
Latino	7	4.7			
Sex					
Male	74	50.0			
Female	74	50.0			
BMI category					
Normal weight	42	28.4			
Overweight	55	37.2			
Obese	51	34.5			

### Weight Bias Internalization

The mean score on the WBIS-M was 5.45 (*SD* = 0.88) on the 7-point Likert scale, suggesting a high level of internalized weight bias in this sample. As depicted in Table 2, there were no significant gender differences in WBI, but differences were present across BMI. Specifically, higher levels of internalization were observed across increasing body weight categories, with the highest mean WBI scores present in adolescents with obesity compared to those at lower body weights.

**Table 2:** Differences in weight bias internalization by weight category and gender.

	Gender								
Weight bias internalization	Males		Females						
	*M*	SD	*M*	*SD*	*t*	*df*	*p*		
	5.49	0.78	5.40	0.97	0.62	145	0.535		

	**Weight category**								
Weight bias internalization	**Healthy weight**		**Overweight**		**Obese**				
	***M***	**SD**	***M***	***SD***	***M***	**SD**	***F***	***df***	***p***

	5.02^a^	0.59	5.40^b^	0.87	5.85^c^	0.92	11.82	2, 147	0.000

^∗^*Note: Superscript letters (a, b, c) indicate significant differences in means between BMI groups. There were no significant interactions between sex and BMI.*

### Associations Between WBI and Eating Behaviors

Table 3 presents results of linear regressions on binge eating severity and eating as a coping strategy as a function of WBI. Binge eating severity accounted for 14% of the variance [*R*^2^ = 0.14, *F*(5,147) = 4.70, *p* < 0.001] and eating as a coping strategy accounted for 39% of the variance [*R*^2^ = 0.39, *F*(5,147) = 17.86, *p* < 0.001] in each dependent variable. WBI was significantly higher among adolescents who had more severe levels of binge eating (as reflected by lower scores; β = -0.18, *p* = 0.036) and eating to cope with distress (β = 0.50, *p* < 0.001).

**Table 3:** Linear regressions on binge eating severity and eating as a coping strategy as a function of weight bias internalization.

	Binge eating severity (lower scores = increased severity)				
Variable	*B*	SE	β	*t*	*p*
Female	-0.34	0.13	-0.23	-2.70	0.008
BMI percentile	-0.01	0.00	-0.18	-1.97	0.051
Age	0.07	0.05	0.12	1.42	0.158
Race (white)	-0.39	0.20	-0.15	-1.91	0.058
WBIS_mean	-0.15	0.07	-0.18	-2.12	0.036

	**Eating as a coping strategy (mean)**				
	***B***	**SE**	**β**	***t***	***p***

Female	0.13	0.07	0.12	1.71	0.090
BMI percentile	0.00	0.00	0.13	1.67	0.097
Age	-0.08	0.03	-0.18	-2.54	0.012
Race (white)	0.12	0.12	0.07	1.03	0.305
WBIS_mean	0.29	0.04	0.50	6.99	0.000

*WBIS, Weight Bias Internalization Scale.*

### Associations Between WBI and Parental Weight-Related Comments

As displayed in Table 4, the regression model (accounting for controls) explained 68% of the variance in WBI (*R*^2^ = 0.68, *F*(16,143) = 16.75, *p* < 0.001). Frequency of weight teasing from peers, but not family members, was associated with higher WBI in adolescents (β = 0.38, *p* < 0.001). Weight-related comments from mothers, but not fathers, also significantly predicted adolescents’ WBI. Specifically, frequency of mothers’ comments about their adolescent’s weight (β = 0.24, *p* = 0.002) and their own weight (β = 0.22, *p* < 0.001) were associated with higher levels of WBI in adolescents. In addition, more frequent dieting efforts to lose weight among mothers (but not fathers) (β = 0.20, *p* = 0.006) was associated with higher levels of WBI.

**Table 4:** Linear regressions on weight bias internalization as a function of weight-based victimization and parental weight-related comments.

	Weight bias internalization				
	*B*	SE	β	*t*	*p*
Variable					
Female	0.07	0.11	0.04	0.62	0.539
BMI percentile	0.00	0.00	0.02	0.28	0.783
Age	-0.04	0.04	-0.06	-0.97	0.335
Race (white)	-0.17	0.17	-0.05	-0.95	0.343
Weight-based victimization by peers (yes/no)	-0.35	0.22	-0.10	-1.58	0.116
Weight-based victimization by family (yes/no)	-0.09	0.13	-0.05	-0.68	0.497
Frequency of peer weight teasing	0.33	0.07	0.38	4.55	0.000
Frequency of family weight teasing	-0.04	0.06	-0.05	-0.72	0.476
Frequency of mother’s comments about adolescent’s weight	0.30	0.09	0.24	3.22	0.002
Frequency of father’s comments about adolescent’s weight	-0.10	0.06	-0.10	-1.63	0.105
Frequency of mother encouraging adolescent to diet	0.07	0.09	0.05	0.73	0.465
Frequency of mother talking about her own weight	0.27	0.07	0.22	3.68	0.000
Frequency of mother dieting	0.21	0.07	0.20	2.81	0.006
Frequency of father encouraging adolescent to diet	-0.03	0.08	-0.03	-0.38	0.702
Frequency of father talking about his own weight	-0.02	0.07	-0.02	-0.36	0.723
Frequency of father dieting	0.12	0.07	0.11	1.76	0.081

## Discussion

The present study provides new insights about internalized weight bias in youth, contributing to the scant literature in this area. In this sample, adolescents exhibited high levels of WBI; the mean WBIS score was higher in comparison to the few existing studies with adolescents in the U.S. and Germany (Roberto et al., 2012; Ciupitu-Plath et al., 2017) using the WBIS measure. Furthermore, we found that adolescents (both girls and boys) endorsed higher levels of internalization across increasing body weight categories. It is unclear why WBI was considerably higher in our sample, as the few comparison studies in this area included both community samples and weight-loss treatment-seeking samples of similarly aged adolescents. This finding highlights the need for additional research to examine levels of WBI in community samples of youth and those seeking different types of weight loss treatment (e.g., behavioral weight loss versus bariatric surgery). Furthermore, we found no gender differences in WBI among adolescents in our study; this is similar to findings by Roberto and colleagues who found no gender differences in WBI among adolescents seeking weight loss surgery, but contrasts with two studies of German youth documenting higher WBI in German girls compared to boys using a modified version of the WBIS (Ciupitu-Plath et al., 2017; Zuba and Warschburger, 2017), as well as the broader literature on WBI in adults which typically shows higher levels of WBI in women compared to men (Pearl and Puhl, 2018).

Our findings suggest that internalized weight bias is important to consider in the context of maladaptive eating behaviors in adolescents. Levels of WBI were significantly higher among adolescents who reported binge eating and eating as a coping strategy. These findings persisted regardless of adolescent BMI, and binge eating severity was higher for girls relative to boys in this sample regardless of internalization. Existing research with adult samples suggests moderate to strong correlations between WBI and binge eating symptoms (Durso et al., 2012b; Pearl et al., 2014; Schvey and White, 2015; Douglas and Varnado-Sullivan, 2016; Palmeira et al., 2016, 2017a), which have also persisted after controlling for BMI (Durso and Latner, 2008). While few studies have assessed adult gender differences, some research has found correlations between WBI and binge eating in both men and women (Boswell and White, 2015). In light of this previous evidence and links between higher WBI and eating pathology observed in our sample, additional research is warranted to better understand the relationship between internalization and maladaptive eating. Given that our sample was comprized of adolescents seeking weight loss, it seems particularly important to determine whether WBI exacerbates eating pathology and ultimately affects weight-related treatment outcomes.

To our knowledge, our study is the first to examine associations between WBI in youth and different sources of weight-based teasing. Results showed that more frequent weight-based teasing from peers, but not from family members, was associated with higher WBI in adolescents. While the reason for this finding is unclear, it may be that the heightened salience and importance of peer relationships and social networks during adolescent development contribute to a stronger impact of weight-based teasing from peers (compared to parents) on adolescents during this time period, which could in turn elevate their levels of internalization in response to these experiences. It will be helpful for future research to examine links between WBI and different sources of weight-based victimization across multiple age groups of youth, to better understand what role peers and family play in WBI for younger and older children. Furthermore, recent work has demonstrated that experiences of weight-based victimization from both peers and family members are associated with adverse emotional and eating-related outcomes, especially for girls (Puhl et al., 2017b). Thus, additional research is needed to clarify whether links between WBI and adverse health indices in youth are attenuated or worsened depending on the perpetrator (family versus peers) of weight-based teasing.

At the same time, our study provides the first evidence of links between parental comments about weight and internalized weight bias in adolescents. While weight-based teasing from parents was not associated with WBI in our study, we found that adolescents’ perceptions of their mothers’ comments about weight and dieting significantly predicted adolescents’ WBI. Specifically, higher levels of WBI in adolescents were associated with a higher frequency of mothers’ comments about their own body weight, comments about their adolescent’s body weight, and encouragement of their adolescent to diet, as well as increased dieting frequency among mothers. These findings remained regardless of adolescents’ gender or body weight. While some previous work has found that maternal comments about weight may play a stronger role in contributing to adverse eating behaviors in youth than comments from fathers (Keery et al., 2005; Neumark-sztainer et al., 2010; Palmeira et al., 2017a), other evidence has found no differences in child outcomes according to parent gender (Gillison et al., 2016). A recent meta-analysis of research examining parental weight talk indicated that critical weight comments from parents and parental encouragement of children to lose weight were associated with poorer physical self-perceptions, more dieting, and dysfunctional eating in children (Gillison et al., 2016). In addition, a recent study of 546 parents found that weight-focused conversations were more common among parents who themselves had recently dieted and perceived their child to be overweight (Winkler et al., 2018). Findings of the present study add new insights to this literature, suggesting that maternal comments about weight may have negative implications for internalization of weight bias and self-blame in youth. It could be that WBI mediates the relationship between poor self-perceptions and eating pathology among children in response to negative parental weight talk; this is an important question for future research. In addition to maternal comments about weight, mothers’ own dieting behaviors were associated with higher WBI in adolescents. Thus, maternal weight-related behaviors, in addition to communication, may be important to study in efforts to better understand WBI in youth. It is unclear whether maternal comments about weight and/or dieting behaviors lead to increased levels of WBI in adolescents, or whether adolescents with higher WBI are more sensitive to and/or aware of their mothers’ weight-related comments and actions. Longitudinal research is necessary to determine the direction and nature of these associations.

### Limitations

The cross-sectional nature of our study prevents conclusions about the directionality of WBI and eating behaviors in youth. The lack of longitudinal research is a limitation in the literature on WBI more broadly (Pearl and Puhl, 2018), and our findings highlight a clear need for experimental and prospective studies to help establish when WBI begins in youth development, it’s progression over time, and the role that it plays in the development and/or reinforcement of unhealthy eating behaviors. Our data relies on self-reports of adolescents, including their reports about parental comments related to weight and dieting. It will be important for future research to examine parent-child dyads to determine whether their perspectives about weight-related communication align. The alpha for the scale assessing coping with stress via eating was lower than anticipated. Measurement in this topic area is a general limitation of this research, and the lack of measures available to assess eating-related coping strategies in youth indicates the need for measure development. As such, the present results should be interpreted with caution as more studies on the relationship between WBV and coping are needed. In addition, our sample was comprized of primarily white adolescents, and given financial costs to families to send their child to a weight loss camp, our sample of adolescents may reflect a higher sociodemographic level than other groups of youth seeking weight loss. Studies are needed to examine the nature and extent of WBI in racially and economically diverse samples of youth across different ages, and in both community and clinical samples of youth. Our sample response rate of 48% suggests that there could be potential response bias; while sample comparisons showed only slight differences in age and geographical location between adolescents who participated in the survey and those who did not, it will be important for future research to improve methods to maximize participation. Finally, our sample consisted of some adolescents at a healthy body weight, who had previously achieved weight loss and were no longer trying to lose weight. We know little about WBI in youth who have obtained a healthy weight status after substantial weight loss, or what role WBI may play during weight loss maintenance in youth. Given that WBI remained high in this group, more research is needed to better understand WBI and its links with eating behaviors as body weight changes, and whether internalization may linger in youth even after they lose weight. Despite these limitations, this study contributes new knowledge to the sparse literature on WBI in youth, points to novel associations with parental factors, and highlights specific areas for future research that can help advance this field of study.

### Clinical Implications

The present findings suggest that it may be useful to inform pediatric health care providers that some youth who seek treatment for weight loss may have elevated levels of WBI, and that WBI may be associated with maladaptive eating behaviors. As more research is conducted on WBI in youth and adolescents, it may be warranted to assess strategies to help reduce WBI. Preliminary studies with adults enrolled in BWL programs have found that clinical intervention approaches (e.g., using cognitive behavioral strategies or acceptance and commitment therapeutic approaches) can be effective in reducing WBI (Pearl et al., 2016; Palmeira et al., 2017b; Levin et al., 2018). It may be useful to extend this research to samples of youth, especially in light of our observed associations with WBI and maladaptive eating behaviors, which could impair treatment efforts in adolescents seeking weight loss. Part of such efforts to reduce WBI could involve addressing parental comments about weight and health, given both the present study’s findings linking WBI to parental weight comments as well as previous evidence demonstrating consistent associations with parent-child weight conversations and adverse health behaviors in youth, such as dysfunctional eating (Gillison et al., 2016). Previous research has demonstrated that parent-child conversations that focus on health behaviors such as nutritious eating and physical activity (rather than body weight) are associated with positive child outcomes, such as healthy eating and increased body satisfaction (Berge et al., 2013, 2015; Gillison et al., 2016). Thus, educating parents (especially mothers) about the potential harmful impact of making comments about their own weight or their child’s weight, and about the benefits of talking about health behaviors rather than weight, could be useful targets for intervention to assess whether these strategies reduce WBI in youth.

## Author Contributions

RP conceptualized and designed the study and drafted the initial manuscript. MH oversaw the data collection, carried out the data analyses, and contributed to writing the manuscript. Both authors revised the manuscript and approved the final manuscript as submitted.

## Conflict of Interest Statement

The authors declare that the research was conducted in the absence of any commercial or financial relationships that could be construed as a potential conflict of interest.
